# Artificial microbial consortia for bioproduction processes

**DOI:** 10.1002/elsc.202100152

**Published:** 2022-04-14

**Authors:** Fabian Mittermeier, Miriam Bäumler, Prasika Arulrajah, José de Jesús García Lima, Sebastian Hauke, Anna Stock, Dirk Weuster‐Botz

**Affiliations:** ^1^ Department of Energy and Process Engineering TUM School of Engineering and Design Chair of Biochemical Engineering Technical University of Munich Garching Germany; ^2^ TUM School of Engineering and Design Technical University of Munich Garching Germany

**Keywords:** artificial consortia, bioproduction processes, cell‐to‐cell interactions, co‐cultivation

## Abstract

The application of artificial microbial consortia for biotechnological production processes is an emerging field in research as it offers great potential for the improvement of established as well as the development of novel processes. In this review, we summarize recent highlights in the usage of various microbial consortia for the production of, for example, platform chemicals, biofuels, or pharmaceutical compounds. It aims to demonstrate the great potential of co‐cultures by employing different organisms and interaction mechanisms and exploiting their respective advantages. Bacteria and yeasts often offer a broad spectrum of possible products, fungi enable the utilization of complex lignocellulosic substrates via enzyme secretion and hydrolysis, and microalgae can feature their abilities to fixate CO_2_ through photosynthesis for other organisms as well as to form lipids as potential fuelstocks. However, the complexity of interactions between microbes require methods for observing population dynamics within the process and modern approaches such as modeling or automation for process development. After shortly discussing these interaction mechanisms, we aim to present a broad variety of successfully established co‐culture processes to display the potential of artificial microbial consortia for the production of biotechnological products.

## 1 INTRODUCTION

Over time, humans have learned to take advantage of natural microbial consortia to produce a variety of products, thus establishing artisanal fermentation processes without knowing about the role of microorganisms. At first, these fermentations mainly focused on foods and beverages, including the production of beer, wine, cheese, or bread. During the last decades, as more knowledge about these procedures was gained, biotechnology became more important and new processes were designed and optimized, often inspired by nature. A recent example of this way of inspiration can be observed in the development of artificial co‐culture processes.

Natural microbial consortia are mostly undefined groups of microorganisms from several different species collaborating, for example, for the degradation of lignocellulosic biomass. This feature is often used in environmental engineering for wastewater treatment or bioremediation [1, 2]. In contrast, artificial consortia or co‐cultures used in bioproduction processes are well‐defined and consist nearly exclusively of two different species or even two different strains from the same species and are specifically designed and optimized for their respective purpose. In this review we will focus on such processes and their advantages and challenges.

Co‐cultivation may provide significant advantages as compared to the cultivation of pure cultures based on several different mechanisms. Process limitations caused by the metabolic burden of gene overexpression, accumulation of toxic and inhibiting byproducts or intermediates or thermodynamic limitations can be evaded by the principles of division of labor or provision and utilization of different substrates, resulting in increased productivity and product yields [3, 4, 5]. Aside from these biological benefits, co‐cultures can also bring advantages due to the reduction of process steps, mainly by only conducting one instead of two or more cultivation processes. Furthermore, the potential integration of biological pre‐treatment or tailored enzyme production into a one‐step process helps to minimize the input of labor or equipment.

Despite these advantages, many challenges yet remain when using consortia instead of monocultures in bioproduction processes, and these challenges have limited implementation on a commercial scale. One of the main challenges is posed by the complex interactions between cells of different organisms. Although many consortia in nature work synergistically, combining non‐compatible strains can provoke competitive or even antagonistic behavior with unfavorable effects on process performance. Thus, the presence of more than one organism deem observation of population dynamics necessary in order to analyze and control growth and interactions within the consortium. Therefore, a variety of methods were developed for resolving population dynamics in co‐cultures [6]. Offline measuring techniques include classical, labor‐intensive methods such as microscopy or plate counting [7] as well as advanced technologies like quantitative PCR or flow cytometry in combination with fluorescent reporter strains or fluorescence in situ hybridization (FISH) [8, 9, 10, 11]. Online analysis of the population distribution can for instance be conducted by online‐flow cytometry or headspace mass spectrometry using volatile signature metabolites of the respective strains [12, 13].

Due to these complex interactions and challenges, the development of co‐culture processes can become difficult and time‐consuming. In order to reduce the experimental efforts, computational methods were introduced. For instance, metabolic modeling approaches were developed to analyze and understand the inter‐species interactions [14]. Other in silico methods can be used to design co‐cultivation processes, for example, by finding ideal division of labor between strains to avoid potential thermodynamic limitations [15]. On the practical side miniaturized and automated high‐throughput methods for parallel cultivation and analysis offer great potential to further minimize the work effort during process development [16].

The number of publications on artificial microbial consortia is constantly increasing. To overcome the aforementioned problems, it is essential to consider many different microorganisms and process designs. This review is intended to summarize the possibilities for bioproduction and, by means of recent examples, to show the current progress in artificial co‐culturing approaches.

## 2 INTERACTION MECHANISMS IN MICROBIAL CONSORTIA

To design synthetic microbial consortia, it is important to understand the possible interaction mechanisms. Although the interaction mechanisms in microbial consortia are complex, they can be classified in few simplified types: mutualism, commensalism, parasitism, competition, amensalism, and neutralism.

In bioproduction processes, a cooperative relationship such as mutualism or commensalism is beneficial. Mutualism describes a strategy in which two or more species benefit from each other, for instance by exchanging metabolites (cross‐feeding). Another widely distributed example of mutualism can be found in syntrophic processes, where methanogenic organisms are dependent from hydrogen formed by their consortium partner, which in exchange profit from hydrogen accumulation being held off [17]. In commensalism, only one member benefits from the other member of the consortium, without further affecting the partner. The production of the vitamin C precursor 2‐keto‐L‐gulonic acid (2‐KGA) by *Ketogulonicigenium vulgare* is, for example, supported by *Bacillus megaterium* by providing nutrients for improved growth and 2‐KGA production of *K. vulgare* without benefits for *B. megaterium* [18]. Parasitism, competition, and amensalism describe negative interactions that are unwanted in most bioprocesses. Parasitism describes a mechanism similar to commensalism. However, in this case the strain, which benefits from the other, also has a negative impact on its partner. In the case of competition, the members of the consortium compete against and thereby negatively affect each other. Competition often takes place due to substrate limitations. In amensalism, only one strain has a negative impact on the other, and neutralism describes a state in which no interaction between microorganisms takes place [17]. In bioprocesses utilizing microbial consortia, the aforementioned types of positive interactions are exploited in several ways. A large number of various interaction mechanisms were investigated and successfully applied using the latter.

### 2.1 Using of co‐cultures for substrate provision

**FIGURE 1:**
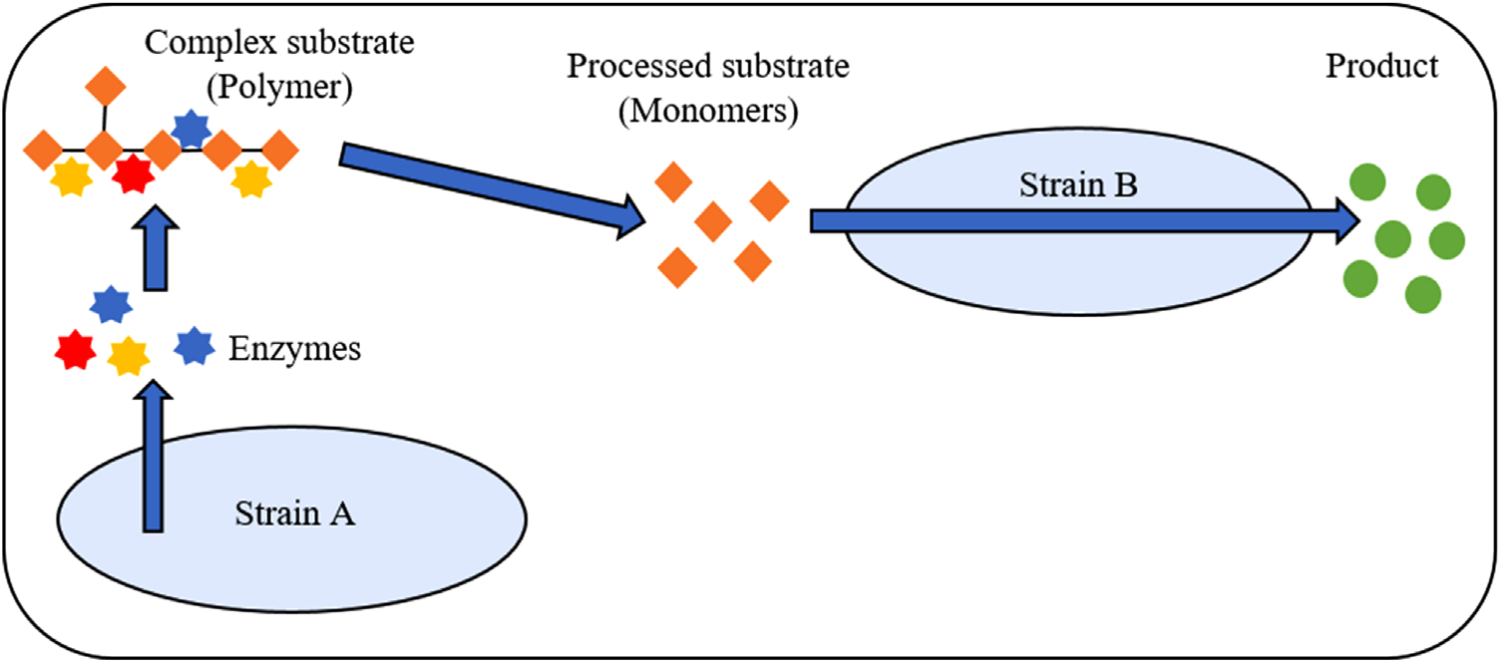
Substrate provision in a co‐culture. Microbial strain A secretes enzymes for the degradation of a complex substrate into easily consumable substrate molecules (e.g., monomers). The processed substrate can then be utilized by strain B for product formation

One big advantage of co‐cultures is the possibility of using low‐cost substrates like starch or lignocellulose for the bioprocesses. Since these substrates are composed of various kinds of linear or branched carbohydrates, their hydrolysis to soluble monomers is a complex process carried out in several steps. Consolidated bioprocesses with co‐cultures integrating enzyme production, hydrolysis and bioconversion into a single process are a promising approach to accomplish this task. Often fungi are used as the enzyme producer (A) as they are well known for their hydrolytic capabilities. The second strain (B) can subsequently utilize the released monomers for growth or conversion into the desired product (Figure 1). This principle was exemplary applied in a co‐culture process with *Trichoderma reesei* (A) and *Ustilago maydis* (B) to produce itaconic acid directly from cellulose [19].

### 2.2 Combining upstream and downstream microbial strains

**FIGURE 2:**
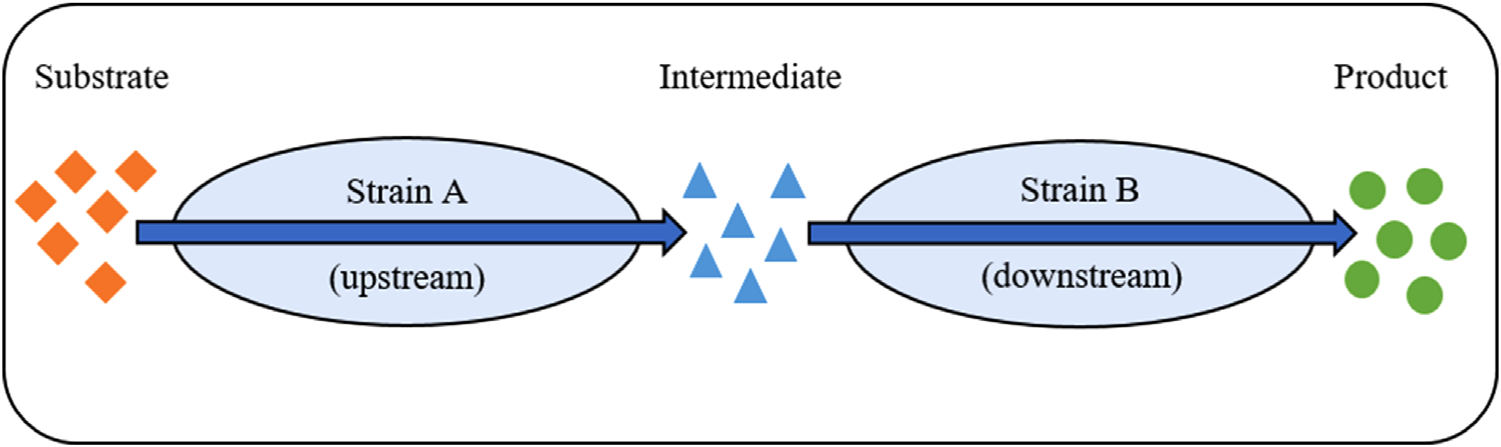
Division of labor between an upstream (A) and a downstream strain (B). The substrate is converted into an intermediate by strain A. Strain B subsequently converts the intermediate into the final product

Another of the many possible mechanisms for using artificial microbial consortia in bioproduction processes is the division of the microbes into upstream and downstream strains. The upstream strain (A) is responsible for taking up the substrate, converting it into an intermediate product, and, finally, secreting it into the medium. The downstream microorganism (B) then produces the product by further processing the intermediate molecule (Figure 2). This separation of production pathways allows a reduction of the metabolic burden for both strains and thus a more efficient production of complex molecules, for example, the flavonoid sakuranetin by two *Escherichia coli* strains [20]. Another example can be found in the improved production of n‐butanol by dividing the NADH‐intensive synthesis into two *E. coli* strains. The upstream strain produces butyrate, which is consequently reduced to butanol in the downstream strain [21].

### 2.3 Using co‐cultures to prevent metabolite inhibition

**FIGURE 3:**
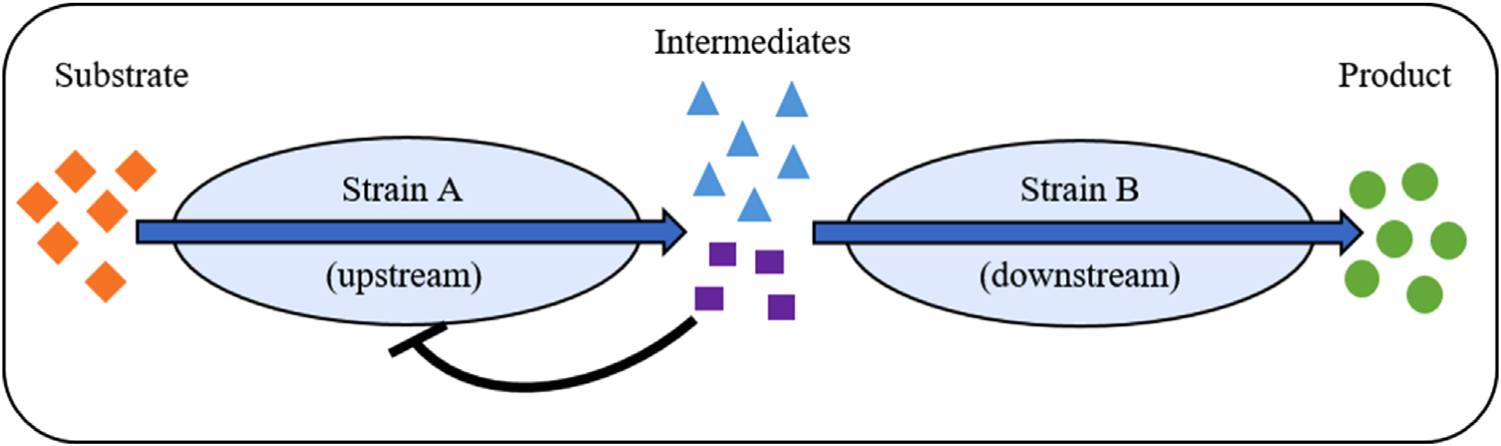
Using a co‐culture to prevent metabolite inhibition. Strain A metabolizes the substrate to an intermediate as well as potentially inhibiting by‐products. Strain B converts the intermediate into the final product while using the by‐product as a carbon source, thus preventing inhibition of strain A

Another advantage of using co‐cultures is the possibility of circumventing feedback inhibition during production. During the bioproduction process, one microorganism (A) might metabolize the substrate to a product, which may be inhibitory to its own growth. However, in this scenario a second microorganism (B) cannot metabolize the substrate, but it uses the product of strain A as a carbon source for growth. Consequently, the concentration of the intermediate product in the medium decreases, thus leading to a reduction of the inhibitory effect on strain A. This type of commensalism results in a higher yield of the final product because strain B removes the intermediate product continuously from the medium in order to synthesize the final product (Figure 3).

This approach has been applied to the overproduction of taxanes, which are the precursor to the anticancer drug paclitaxel. A synthetic mutualistic co‐culture was formed in this specific approach where parts of the whole pathway were separated in different species cultured together for fast production of taxadienes in *E. coli* and improved oxygenation by *Saccharomyces cerevisiae*. Aside from the intermediate, the upstream *E. coli* forms acetate which acts inhibiting on its growth. This is however evaded by the utilization of acetate as substrate for the downstream yeast converting taxadienes to taxanes [22]. Another widely spread example is posed by consolidated bioprocesses with integrated hydrolysis of (ligno‐)cellulosic biomass. The presence of monosaccharides leads to product inhibition of the cellulolytic enzymes. This effect is avoided because the monomeric sugars serve as carbon source for the second strain, so no accumulation takes place [23].

### 2.4 Co‐cultivation of photosynthetic and heterotrophic microorganisms

**FIGURE 4:**
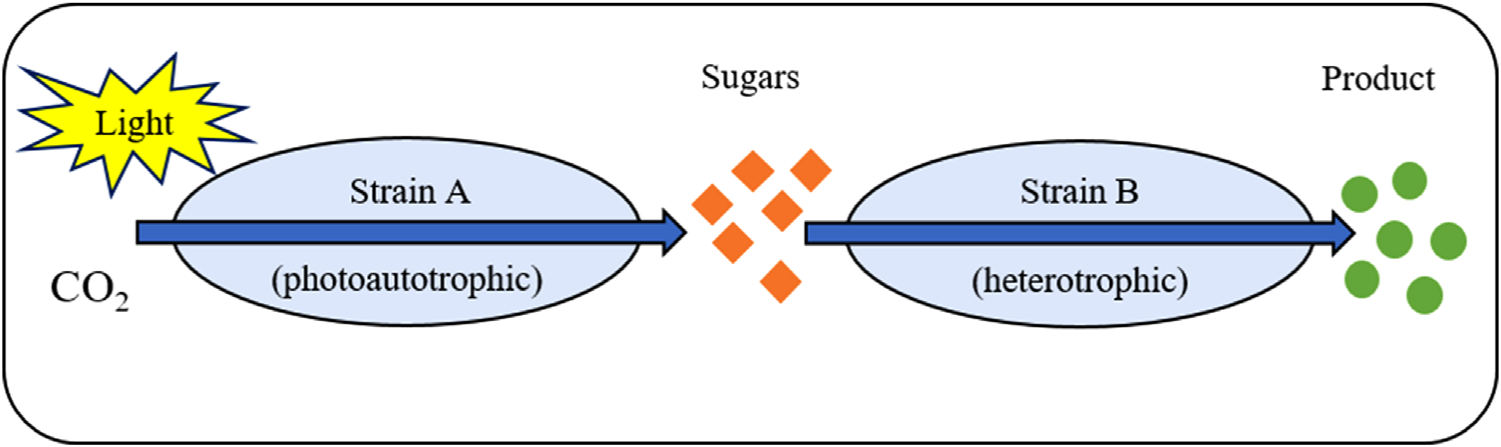
Co‐cultivation of photosynthetic and heterotrophic organisms. The photoautotrophic strain uses carbon dioxide and light to produce sugar molecules. The heterotrophic strain subsequently utilizes these as substrate for the synthesis of a defined product

Since global climate change has become more prominent over the years, researchers have been focusing on the use of solar energy to synthesize target products. Photoautotrophic microorganisms in particular have attracted attention regarding the production of biofuels and chemicals directly from inorganic carbon dioxide (CO_2_). The photoautotrophic bacterium uses sunlight to fixate CO_2_ through the Calvin cycle and produces soluble sugar molecules, which are secreted into the medium. The heterotrophic bacterium uses this substrate and synthesizes the desired product (Figure 4). This type of interaction represents commensalism because only one consortium member benefits from the other, but has no negative effect on the other (with the exception of shading in the microbial suspension). The mechanism described can be used in the production of 3‐hydroxypropionic acid (3‐HP). This product has a wide range of applications in the chemical sector, for example, in the production of chemicals like biodegradable plastic poly‐3‐hydroxypropionicacid, or as a food additive [24].

### 2.5 Co‐cultivation of aerobic and anaerobic microorganisms

**FIGURE 5:**
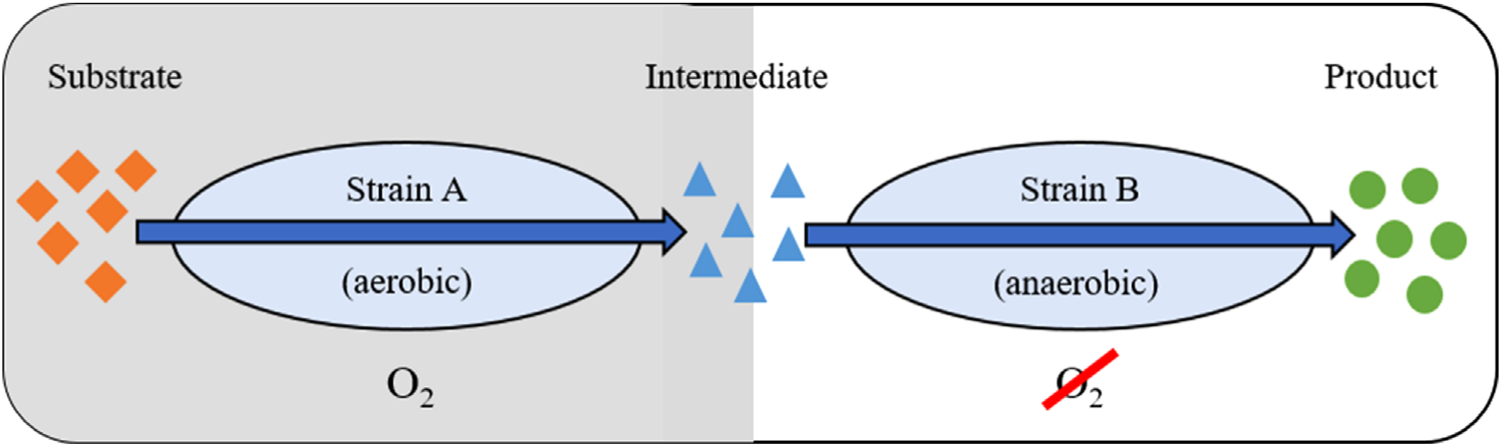
Co‐cultivation of aerobic and anaerobic organisms. The aerobic strain A synthesizes an intermediate product while depleting dissolved oxygen by respiration, thereby creating an oxygen‐free environment for the anaerobic strain B, which converts the intermediate into the final product

The cultivation of anaerobic microorganisms for bioproduction processes is often associated with additional costs stemming from the addition of reducing agents, or flushing with nitrogen to ensure anaerobic conditions in the bioreactor. Co‐culturing aerobic with anaerobic microorganisms may serve as an alternative for removing the oxygen from the medium. The consumption of oxygen by the aerobic consortium member enables the growth of the anaerobic partner and the production of the target molecule. Co‐cultivation of aerobic with anaerobic microorganisms can thus decrease the total cost of acetone‐butanol‐ethanol (ABE) fermentation and increase the productivity compared to the conventional ABE fermentation [25] (Figure 5).

One recent study took advantage of this mechanism to produce lactic acid from cellulose using a membrane reactor, which ensured locally defined aeration through an oxygen permeable membrane. During cultivation, the aerobic consortia member *T. reesei* forms a biofilm on the surface of the membrane prevailing anaerobic condition in the bulk phase. At the same time, this fungus produces and secretes enzymes responsible for the breakdown of lignocellulosic material into glucose as substrate for the anaerobic partner, *Lactobacillus*. While the anaerobic cells are then shielded from the oxygen, lactic acid can be synthesized from the monosaccharides [26]. Another research group applied this method in order to produce lignocellulosic ethanol. In the latter work, a symbiotic consortium was established consisting of the anaerobic cellulolytic mesophilic *Clostridium phytofermentans* and a cellodextrin fermenting yeast (*Candida molischiana* or *S. cerevisiae*) for ethanol production. The aerobic yeast protected the anaerobic *C. phytofermentans* by removing the oxygen. In return, the anaerobic strain hydrolyzed cellulose to soluble cellodextrins, which can be used by the yeast for ethanol production [27].

## 3 SYNTHETIC BACTERIAL CONSORTIA

### 3.1 *Escherichia coli* consortia

*E. coli* has become one of the most widely used microorganisms in the biotech sector due to its ability to grow quickly with cheap carbon sources. *E. coli* is used to synthesize a wide variety of products. Additionally, recent advances in metabolic engineering and systems biotechnology have greatly advanced research on microbial biosynthesis in general. Given that the implementation of microbial consortia is assuming an increasingly important role, it seems clear that the use of single‐species *E. coli* consortia is also on the rise. Generally, the mechanism of choice when using *E. coli* co‐culture is the upstream‐strain/downstream‐strain mechanism, thus splitting complex production pathways and reducing the metabolic burden for each strain.

For example, Li et al. showed how to efficiently produce the complex natural product rosmarinic acid (RA) using an *E. coli* co‐culture in shake flasks. RA is generated by a condensation reaction of the two precursors caffeic acid (CA) and salvianic acid (SSA). In first co‐culture experiments consisting of only two *E. coli* strains, the synthesis of one of the main precursors was performed using the upstream strain, whereas the production of the second precursor and the final product took place in the downstream strain. As both precursors compete for the carbon flux from the tyrosine biosynthetic pathway, the production of the precursors was afterwards divided to two different strains. By further separating the synthesis by implementing a third *E. coli* strain, CA and SSA were produced by two different upstream strains and the final product RA, generated by the third (downstream) strain, was produced with a 38‐fold increased final concentration of 172 mg L^–1^ compared to a monoculture of *E. coli* [28]. Zhang et al. tested two different co‐cultivation strategies in order to improve the production of muconic acid from glycerol using engineered *E. coli* strains. This co‐culture sought to overcome a slow reaction step by either adding a second strain harboring the respective reaction, or by splitting the production pathway and increasing the share of the downstream strain. Using the latter strategy, they achieved a final concentration of 2 g L^–1^ muconic acid from glycerol in a batch bioreactor [29]. In the same year, the same research group produced muconic acid using an *E. coli* co‐culture growing on a glucose/xylose mixture, with the upstream strain consuming only glucose and the downstream strain depleting xylose. They achieved a highly efficient utilization of a sugar mixture and a final concentration of 4.7 g L^–1^ muconic acid [30], which is significantly higher than in any previous reports [31, 32, 33]. Other acids, whose production yield could be improved by co‐cultures of upstream and downstream strains were, for example, caffeoylmalic acid or the phenolic protocatechuic acid, both produced with glucose [34, 35].

Recently, scientists have also focused on the production of flavonoids with bacteria. Flavonoids can be found naturally in fruits, vegetables, wine or tea, etc. This product class is particularly interesting given its high pharmaceutical and nutritional value. Many flavonoids exhibit antioxidant and anti‐inflammatory activity and act as anticancer agents [36, 37]. It has also been found that flavonoids can reduce the risk of coronary heart disease [38]. By virtue of the complex production pathways, research groups are developing *E. coli* co‐cultivation approaches used to produce a wide variety of flavonoids. The following paragraphs describe some of the recent findings in this field.

In one study by Wang et al., the flavonoid sakuranetin was produced using two *E. coli* strains from glucose as a carbon source. The synthesis of the molecule was divided into the production of *p*‐coumaric acid from the central metabolism with the upstream strain (11 biosynthetic steps), and the conversion of *p*‐coumaric acid into sakuranetin by way of the downstream strain (six biosynthetic steps). In the end, 79.0 mg L^–1^ sakuranetin was obtained in a fed‐batch process, which is (to the authors’ knowledge) the highest concentration ever obtained with de novo biosynthesis in *E. coli* [20].

Thuan et al. worked on the production of apigetrin. In contrast to the previously described mechanism, these researchers added *p*‐coumaric acid to the co‐culture, so the upstream *E. coli* strain converted the acid to apigenin (four biosynthetic steps), from which the downstream *E. coli* strain synthesized apigetrin via a glycosyltransferase reaction. They achieved a final concentration of 16.6 mg L^–1^ apigetrin, which was 2.5 fold higher than the results achieved with an *E. coli* monoculture [39].

The microbial production of flavonoids might also reach a very high level of complexity. This was shown first by Jones et al., who chose an experimental approach together with a computational optimization in order to increase final product concentrations in the microbial production of flavan‐3‐ols afzelechtin and catechin. A malonyl‐CoA requiring an upstream *E. coli* strain (phenylpropanoic acids to flavanones) was combined with a NADPH requiring a downstream *E. coli* strain (flavanones to flavan‐3‐ols). The maximum concentration achieved was 41 mg L^–1^ flavan‐3‐ols (34 mg L^–1^ in the scale‐up) by artificially adding *p*‐coumaric acid as a precursor [40]. This approach was extended to a polyculture of four *E. coli* strains by adding a strain for the production of *p*‐coumaric acid and caffeic acid from glucose, on the one hand, and adding a strain for production of anthocyanidin‐3‐O‐glucosides from flavan‐3‐ols on the other hand, resulting in the de novo production of flavan‐3‐ols and anthocyanidin‐3‐O‐glucosides solely from glucose [41]. The other flavonoids produced in *E. coli* co‐cultures are icariside D2 [42] and resveratrol [43].

Curcuminoids, similar to flavonoids, also exhibit antioxidant, anticancer, and antitumor activity. Curcuminoids were produced in an *E. coli* co‐culture in which one strain was used to produce *p*‐coumaric acid from glucose, and the other strain was used for its conversion to the final curcuminoid product in a lab‐scale bioreactor. After various optimization steps, Fang et al. achieved a final concentration of 6.28 mg L^–1^ bisdemethoxycurcumin. Nevertheless, the final concentrations were still twice as high with an alternatively implemented two‐stage, single‐species *E. coli* process [44]. Two years later, Rodrigues et al. produced 15.9 mg L^–1^ of curcumin and a total curcuminoid concentration of 41.5 mg L^–1^ using an *E. coli* co‐culture. These concentrations are, to the authors’ knowledge, the highest concentrations of curcumin/curcuminoids ever obtained by means of microbial production [45].

Saini et al. designed a consortium of two *E. coli* strains to circumvent carbohydrate catabolite repression (CCR) during n‐butanol production from glucose and xylose, which account for the majority of monosaccharides in plant biomass. CCR prohibits the uptake of less preferable carbon sources like xylose in presence of glucose, resulting in incomplete utilization of the available substrates. By applying a glucose‐selective and a xylose‐selective strain they achieved a n‐butanol concentration of 4 g L^–1^ or 50% of the theoretical yield. Furthermore, the highly NADH‐intensive n‐butanol synthesis pathway was distributed into the two strains with the glucose‐selective strain producing and secreting butyrate, which is subsequently converted by the xylose‐selective strain to butanol with the byproduct acetate, which is necessary for butyrate formation of the former strain. Using this strategy a final n‐butanol concentration of 5.2 g L^–1^ was achieved, accounting for 63% of the theoretical yield [21].

### 3.2 *Clostridium* consortia

*Clostridia* are also well‐known production hosts in the biotech sector. However, their cultivation is more demanding than the cultivation of *E. coli* because *Clostridia* are obligate anaerobes. *Clostridium* co‐cultures can be used to improve the production of butanol (and butanol derivatives) or hydrogen and fatty acids, or to simply expand the substrate spectrum in a production process [46].

*Clostridia* are often used for ABE fermentation. Co‐cultures are introduced to maximize the resulting yield of acetone, butanol, and ethanol. In ABE fermentation with *Clostridium* consortia, scientists have focused on numerous different production aspects using a wide variety of bacterial strains and substrates. Many scientists work with a co‐culture in which one strain has lytic properties on the substrate, and the other (non‐lytic) strain exhibits high activity in producing ABE. For example, Kiyoshi et al. produced butanol from rice straw by culturing *C. thermocellum* and *C. saccharoperbutylacetonicum* [47]. Furthermore, Li et al. used *C. beijerinckii* and *C. tyrobutyricum* to produce butanol from cassava starch [48]. Using *Clostridium* co‐cultures, 5‐fold increased amounts of butanol of 3.94 g L^–1^ compared to the wild‐type consortia can also be produced from lignocellulose [49] and 7.9 g L^–1^ butanol from crystalline cellulose [50]. Wen et al. worked on the production of butanol from alkali extracted deshelled corn cobs in a *Clostridium*‐only consortium consisting of *C. cellulovorans* and *C. beijerinckii* [51].

Islam et al. worked with *Clostridium* co‐cultures, with the aim to produce biohydrogen. Using *C. thermocellum* and *C. thermosaccharolyticum* growing on sweet sorghum stalks, they obtained a hydrogen production of 5.1 mmol g_substrate_^–1^. In this case, *C. thermocellum* was breaking down cellulose and hemicellulose to soluble sugars that the higher hydrogen‐yielding *C. thermosaccharolyticum* could consume [52]. Geng et al. worked with a similar co‐culture that utilized starch as a substrate for *C. thermocellum* and *C. thermopalmarium* [53].

Recent publications have shown that *Clostridia* can be used to further process CO or the CO fraction of synthesis gas (syngas) in order to produce useful chemicals from (waste) gas. In general, carboxydotrophic bacteria, like *Clostridium*, exhibit a higher product selectivity than chemical catalysts. Diender et al. worked with a co‐culture consisting of *C. autoethanogenum* and *C. kluyveri* to produce medium‐chain fatty acids (butyrate and caproate) and higher alcohols (butanol and hexanol) from syngas. During the co‐cultivation process, *C. autoethanogenum* utilized the CO to produce acetate and ethanol, which were converted by *C. kluyveri* to form medium chain fatty acids by chain elongation [54]. Further investigation showed that the solventogenic activity of the co‐culture was able to be stimulated with the addition of hydrogen or acetate [55]. Benito‐Vaquerizo et al. further suggested optimizing the production of medium‐chain fatty acids by adding succinate, or by increasing the ethanol production of *C. autoethanogenum* by blocking either acetaldehyde dehydrogenase or formate dehydrogenase (ferredoxin) activity [56]. Alternatively, Richter et al. used a co‐culture of *C. ljungdahlii* and *C. kluyveri* to demonstrate the production of butanol, hexanol, and traces of octanol from syngas [57]. To gain a better insight in the composition of such co‐cultures, two specific 23S rRNA oligonucleotide probes, ClosKluy and ClosCarb, were designed for the monitoring of *C. kluyveri* and *C. carboxidivorans* in co‐culture, respectively [10]. Building on that, Bäumler et al. showed that the individual growth behavior and product formation of each strain in the co‐culture contributed significantly to the efficient carbon monoxide conversion and formation of butanol and hexanol in the synthetic co‐culture [11]. This has all clearly demonstrated the need for an unbiased individual measurement of the cell concentrations in a co‐culture to identify suitable process conditions for improved product formation. In all of the applications mentioned, *C. kluyveri* acted as the chain elongator [36]. In order to also be able to process CO_2_ in the aforementioned co‐cultures, Haas, et al. implemented a CO_2_ electrolyzer to produce CO from CO_2_ and H_2_O [58].

### 3.3 Mixed bacterial consortia

Scientists today are working not only with pure *E. coli* or *Clostridium* co‐cultures, but also with mixed bacterial consortia. In addition to those already mentioned with respect to bacteria, this allows the use of mechanisms that have not been widely investigated before. Additionally, the specific strengths of every single chosen species can be exploited. Eventually, this will lead to both the production of a higher number of various products and the ability to use an extended variety of substrates.

One famous product of mixed bacterial consortia is 2‐keto‐L‐gulonic acid (2‐KGA), a precursor of vitamin C. Conventionally, vitamin C is produced in a two‐step fermentation process using three different bacterial species, in which case *Gluconobacter oxydans* produces L‐sorbose from D‐sorbitol, and *Ketogulonicigenium vulgare* forms 2‐KGA from L‐sorbose accompanied by *Bacillus* spp. for the promotion of growth and production efficiency by releasing active substances. In 2016, Wang et al. implemented a one‐step fermentation using only two different species instead of the two‐step fermentation. After optimizing and engineering the co‐culture consisting of *G. oxydans* and *K. vulgare* by deleting genes involved in the sorbose metabolism of *G. oxydans*, therefore alleviating competition between both strains, their final yield was comparable to the conventional 2‐step fermentation [59]. Also, Ma et al. found a way to achieve conventional yields with a one‐step fermentation process, but, instead of applying a two‐species co‐culture, they worked with three species: *G. oxydans*, *K. vulgare* and *Bacillus endophyticus*. The co‐existence of *G. oxydans* together with *B. endophyticus* supplied additional nutrients, hence promoting growth and the 2‐KGA production of *K. vulgare*. Simultaneously, the growth of the both first strains was decreased due to substrate competition with *K. vulgare*, resulting in increased 2‐KGA production [18]. Many approaches exist for further enhancing 2‐KGA yield. These include, for example, the implementation of a three‐stage temperature control strategy to fulfill the needs of growth and sporulation of *Bacillus megaterium*, growth and metabolism of *K. vulgare* and the enzyme activity of the L‐sorbitol dehydrogenase as there are different temperature optima [60] or the regulation of cell growth of *B. megaterium* with lysozyme for release of intracellular components for increased 2‐KGA production by *K. vulgare* [61].

Aside from the *Clostridium* co‐cultures described above, mixed species consortia can be applied in ABE fermentation processes. For example, Cui et al. implemented a co‐culture for increased butanol production using the aerobic‐anaerobic‐mechanism. During the fermentation, *Bacillus subtilis* depleted oxygen to enable the growth of the obligate anaerobe *Clostridium beijerinckii*. The key advantage of this approach is the dispensable genetically modification of *C. beijerinckii*. After optimizing the process, 6.4 g L^–1^ butanol and 3.5 g L^–1^ butyrate could be obtained, proofing a successful co‐culture with feeding and protection between both strains [62]. A similar aerobic‐anaerobic co‐culture was developed by Tran et al., who worked with *Bacillus subtilis* in a co‐culture with *Clostridium butylicum* by using starch as substrate for ABE production [63]. Later on, Said et al. implemented a consortium of *Bacillus toyonensis* and *Stenotrophomonas rhizophila* to reduce the oxygen demand in the aerobic fermentation of palm oil mill effluent (POME). The introduction of the aforementioned co‐culture (in which *B. toyonensis* had proteolytic and cellulolytic properties, and *S. rhizophila* had lipolytic properties) significantly decreased the oxygen demand together with increased methane production [64].

Another approach is a co‐culture using organisms of the genus *Geobacter* taking advantage of *Geobacter*’s ability to perform direct interspecies electron transfer (DIET) [65]. This mechanism relies on electrically conductive pili or outer membrane cytochromes [66]. By forming of pili, a bridge between electron donor and electron acceptor for electron transfer is created [67]. *Geobacter sulfurreducens* acting as electron donor provides electrons for *C. pasteurianum* as electron acceptor to favor the formation of reduced products. Glycerol served as fermentation substrate for *C. pasteurianum* and acetate as electron donor for *G. sulfurreducens*. The batch experiments performed in serum bottles showed a clear metabolic shift towards improved 1,3‐propanediol and butyrate production caused by the electron transfer, while the concentrations of ethanol and butanol decreased. The authors would like to point out that this effect opens possibilities for controlling product specificities in mixed cultures [68].

## 4 SYNTHETIC FUNGAL CONSORTIA

### 4.1 Yeast consortia

A common way of producing bioethanol from agricultural residues is the hydrolysis of the plant material followed by fermentation with yeasts, mainly *S. cerevisiae*. However, this species is originally not able to metabolize pentoses such as xylose [69]. Therefore, co‐culturing *S. cerevisiae* with pentose‐converting yeasts is a promising approach to achieve complete utilization of hydrolyzed plant biomass.

Using *S. cerevisiae* together with *Spathaspora passalidarum*, Farias et al. increased the ethanol production in shake flasks from 50% sugarcane molasse and 50% hemicellulosic bagasse hydrolysate up to 30.2 g L^‐1^ with depletion of all available sugars [70].

Hashem et al. cultivated a consortium of *S. cerevisiae*, *Pichia barkeri*, and *Candida intermedia* on hydrolyzed rice waste, resulting in almost complete consumption of the sugars contained and an ethanol yield of 0.167 g g^–1^ or 96% of theoretically possible conversion, compared to 74.3 – 80.3% with respective monocultures using a 7 L bioreactor [71]. Similarly, Pathania et al. fermented hydrolyzed apple pomace waste with *S. cerevisiae* and *Scheffersomyces stipitis* to produce 34.46 g L^–1^ ethanol, which was further improved by immobilization to a maximum of 44.46 g L^‐1^ [72].

Sunwoo et al. used a consortium of *S. cerevisiae* and *Pichia angophorae* to obtain ethanol from seaweed hydrolysate primarily consisting of glucose, galactose, and mannitol. Glucose can be utilized by both strains, whereas galactose and mannitol are solely converted by *S. cerevisiae* or *P. angophorae*, respectively. Using this consortium with a preceding substrate adaption phase, a final ethanol yield of 0.45 g g^–1^, and a conversion rate of 94% was achieved after 120 h [73]. Verhoeven et al. chose to genetically engineer three different strains of *S. cerevisiae* to selectively ferment glucose, xylose, and arabinose, respectively. In repeated anaerobic batch cultivation they achieved a three strain consortium demonstrating stable fermentation performance. [74].

In order to enable direct ethanol production from biomass, yeasts can be recombinantly equipped with hydrolytic enzymes for simultaneous saccharification and fermentation. Such a co‐culture was created with a recombinant *S. cerevisiae* strain expressing three cellulases and a recombinant *Pichia pastoris* strain expressing two xylanases [75]. This way, an ethanol yield of 0.42 g g^–1^ and a final concentration of 32.6 g L^–1^ was achieved in shake flasks after 70 h directly from wheat straw. A consortium established by Qadir et al. consisting of a *S. cerevisiae* strain natively producing cellulases and pectinase and the pectinase producer *Geotrichum candidum* was immobilized on corn cob pieces and produced 17.89 U ml^–1^ pectinase activity with orange peels. [76].

Aside from genes coding for hydrolytic enzymes, genes for heterologous production of complex molecules can be introduced into yeast cells, which can be applied in co‐cultures using the mechanism of pathway splitting. Liu et al. produced the pharmaceuticals lovastatin and monacolin J with either methanol [9], or ethanol [77] as carbon sources applying a co‐culture of two engineered *P. pastoris* strains overexpressing genes from *Aspergillus terreus*. In bioreactor experiments concentrations of 593.9 mg L^–1^ monacolin J and 250.8 mg L^–1^ lovastatin could be reached with methanol and 2.2 g L^–1^ monacolin J with ethanol. A pathway splitting co‐culture platform for the production of various flavonoids with different strains of *S. cerevisiae* was developed by Du et al. [78]. Combining a naringenin‐producing upstream strain and a specific downstream strain enables the flexible production of six flavonoids including delphinidin.

### 4.2 Filamentous fungal consortia

One of the main challenges in sustainable conversion of plant materials to, for example, high‐value chemicals or biofuels is posed by its recalcitrance, hindering its utilization by microorganisms. Therefore, filamentous fungi are often used for the production of enzymes for hydrolysis of lignocellulosic biomass to release easily available compounds for other organisms. These processes can be further improved economically by producing the enzymes needed on‐site using fungal consortia with complementing enzyme activities [79]. For example, it has been shown that the widely used ascomycetes *T. reesei* and *Aspergillus niger* complement each other in the degradation of cellulose. *T. reesei* forms high cellobiohydrolase activity, while *A. niger* produces higher β‐glucosidase and endoglucanase activity [80]. By co‐cultivating these strains in a 3 L bioreactor with a complex medium containing cellulose, filter paper activity and volumetric enzyme productivity was doubled compared to an *A. niger* monoculture [81]. Similarly, Kolasa et al. were able to highly enhance cellulolytic enzyme activity and hydrolysis efficiency of pre‐treated wheat straw by combining *T. reesei* with different *Aspergillus* strains [82].

Since the interaction mechanisms between differing fungal strains are often poorly characterized, a statistical approach can be used to find the optimal combination for a consortium. Using a Taguchi design, Lin et al. evaluated 32 cellulolytic consortia built from combinations of six different fungal strains [83]. The final optimized consortium consisting of *Trichoderma* sp. T‐1, *Phanerochaete chrysosporium*, and *A. oryzae* A‐4 achieved a 26.98% higher yield of reducing sugars from wheat straw, when compared to *Trichoderma* sp. T‐1 alone.

Consortia can also be used to simultaneously digest cellulose and hemicellulose. For this purpose, Zhao et al. co‐cultured a cellulase‐secreting *Aspergillus flavus* strain with a xylanase‐secreting *Aspergillus penicillioides* [84], increasing the reducing sugar concentration twofold compared to using just the cellulase‐producing strain, achieving a final sugar concentration of 29.8 g L^–1^, compared to 14.8 g L^–1^, after 56 h incubation. The enzymatic hydrolysis step was carried out separately after the fungal co‐culture in solid‐state fermentation (SSF) on wheat bran. Despite that, the authors were able to show that the use of the co‐culture led to comparable yields as with respective monocultures with subsequent enzymatic hydrolysis using a mix of culture supernatants.

Aside from on‐site enzyme production and hydrolysis, fungal consortia can also be used in consolidated bioprocesses, where sugars released from lignocellulosic biomass by these hydrolytic enzyme systems are subsequently converted into products of value. Bastos et al. used a consortium of *A. niger* and *T. reesei* in a SSF enabling simultaneous saccharification and citric acid production from sugarcane bagasse with the productivity being comparable or even higher than with the respective monocultures while significantly reducing the labor input [85]. Pursuing a similar approach, Scholz et al. co‐cultured *T. reesei* with two different production specialists, *Rhizopus delemar* and *Rhizopus oryzae*, to produce fumaric acid and lactic acid, respectively, in shake flasks [86].

### 4.3 Mixed yeast – filamentous fungi consortia

Artificial consortia of yeast and filamentous fungi have been proposed mainly with the goal of establishing consolidated bioprocesses (CBPs), where enzyme production, hydrolysis and fermentation take place in a single process. In these consortia, the fungus’ role consists of substrate provision by enzyme secretion and hydrolysis of lignocellulosic biomass into respective monomers, which subsequently can be converted to specific products by the yeast. Additionally, the conversion of the monomers helps to prevent potential product inhibition of the fungal enzymes [80].Thus, CBPs have potential to establish lower process costs, for example, for biofuels or bulk chemicals.

Intasit et al. established a consortium of lignocellulosic and oleaginous fungus *Aspergillus tubingensis* and oleaginous yeast *Yarrowia lipolytica* to produce biodiesel from palm empty fruit bunch under non‐sterile conditions, with a maximum lipid concentration of 165 mg g^–1^ [87].

Schlembach et al. established a CBP to produce the platform chemical itaconic acid from cellulose, using cellulolytic *T. reesei* with *U. maydis* as the itaconic acid producer [19]. They achieved a yield of 0.16 g g^–1^ with a productivity of 0.07 g L^–1 ^h^–1^ during a fed‐batch cultivation. Another example is the production of fructo‐oligosaccharides (FOS) via a consortium of *Aureobasidium pullulans* and *S. cerevisiae* [88]. Productivity was increased from 4.9 to 5.9 g L^–1^ h^–1^ compared to the monoculture of *A. pullulans*. In this case the instant removal of released glucose avoided enzyme inhibition and increased the purity of the product.

## 5 MIXED CONSORTIA

### 5.1 Artificial bacterial‐fungal co‐cultures

Co‐cultures composed of bacteria and fungi or yeasts are investigated to produce a wide range of products, among them alcohols and organic acids [23]. These consortia of bacterial and fungal cells are often used in consolidated bioprocesses to convert renewable substrates such as biomass into chemicals.

Ho et al. were able to develop a bioprocess with recombinant *Bacillus subtilis* and *Kluyveromyces marxianus* that produces ethanol from cellulose. *B. subtilis* was recombinantly equipped with eight cellulose‐hydrolyzing enzymes from *Clostridium thermocellum*, while *Kluyveromyces marxianus* heterologously produced the β‐glucosidase enzyme from *Neocallimastix* sp. W5. The genetic modifications of the kefir yeast enabled effective saccharification and increased ethanol production 6‐fold ‐compared to a *K. marxianus* monoculture or a co‐culture with the kefir yeast wild‐type [89].

A consortium designed by Wang et al. consisting of *S. cerevisiae* and an ethanologenic *E. coli* strain engineered to not utilize glucose was used to convert pretreated sugar cane bagasse slurry to ethanol. With the yeast utilizing glucose and the *E. coli* fermenting xylose, an ethanol concentration of 24.9 g L^–1^, corresponding to 70% of the theoretical yield, was achieved within less than 30 h [90]. Qian et al. developed a co‐culture of *S. cerevisiae* and engineered *E. coli* carrying an alcohol dehydrogenase and pyruvate decarboxylase from *Zymomonas mobilis* achieving an ethanol yield of 0.49 g g^–1^ or 96.1% conversion from softwood hydrolysate [91]. Another consolidated bioprocess is the mixed cultivation of *C. phytofermentans* with *S. cerevisiae* in a 500 ml bioreactor by Zuroff et al. for ethanol production from cellulose. This mixed aerobic‐anaerobic process is enabled by controlled oxygen transfer into the reactor enabling the yeast to protect the anaerobic *Clostridium* from the introduced oxygen in return for soluble sugars released by the hydrolytic enzymes produced by the anaerobic bacterium. Additional cellulase led to a further increased conversion of cellulose resulting in 22 g L^–1^ ethanol, compared to 6 and 9 g L^–1^ in *C. phytofermentans* and *S. cerevisiae* monocultures, respectively [27].

A study by Tri and Kamei demonstrated the production of butanol from cellulose by an anaerobic consortium of *Clostridium saccharoperbutylacetonicum* and the white‐rot fungus *Phlebia*. The co‐cultivation increased saccharification, and combined with genetic inhibition of ethanol formation, final concentrations of 3.2 g L^–1^ butanol were achieved on a small scale (100 ml) [92].

Consolidated bioproduction of isobutanol from pretreated corn stover was demonstrated with *T. reesei* and *E. coli*. With a comprehensive mathematical model of the consortium to optimize key parameters, an overall concentration of 1.88 g L^–1^ isobutanol was achieved in shake flasks. Additionally, dynamics within the co‐culture were analyzed in detail for further stabilizing and tuning the consortium [93].

Shahab et al. used a bacteria‐fungus consortium to produce lactic acid from lignocellulosic substrates. *T. reesei* fungi are responsible for the secretion of cellulolytic enzymes while the facultative anaerobic *Lactobacillus pentosus* was used to produce lactic acid. Concentrations of 20 g L^–1^ lactic acid could be achieved in a spatially structured biofilm using pretreated wood and hydrolysate as substrate in a fed‐batch process. In another co‐cultivation study using microcrystalline cellulose as substrate, they were able to produce 34.7 g L^–1^ lactic acid in a batch process in a 500 ml biofilm membrane reactor with a sequential inoculation scheme of *T. reesei* and *L. pentosus* [26].

Liu et al. constructed a consortium of *Pseudomonas putida* and *S. cerevisiae* for the production of polyhydroxyalkanoates (PHA) from xylose. The substrate was converted to lactic acid by *S. cerevisiae*, which is a preferable substrate of *P. putida*. By choosing an ideal inoculation ratio of *P. putida* versus *S. cerevisiae* of 1:10, they were able to increase the PHA concentration tenfold compared to *P. putida* monoculture, which could only convert small amounts of xylose. In addition, sedimentation of the cells after the end of cultivation was improved, simplifying product purification [94].

Recent studies have shown that the modular approach, as already described for bacterial consortia, can also play an important role in fungal‐bacterial co‐cultures. Zhou et al. designed a stable co‐culture of engineered *E. coli* and *S. cerevisiae* to produce taxanes, a precursor for anticancer drugs, from xylose. This monosaccharide was chosen as main substrate instead of glucose in order to avoid ethanol production by the yeast as it cannot utilize xylose. *E. coli* was genetically equipped to produce taxadienes, which were subsequently oxygenated by the *S. cerevisiae* cells. Acetate, a byproduct of *E. coli*, serves as the carbon source for the yeast, which also brings the benefit of evading acetate inhibition of the bacterial cells. An optimized inoculum ratio of *S. cerevisiae* to *E. coli* (40:1) enabled the production of taxanes. Subsequently, this strategy was adapted to the formation of other compounds like tanshinone precursors and functionalized sesquiterpenes [22]. Another modular approach for using these organisms was developed to produce narginine. *E. coli* was modified to efficiently convert D‐xylose into L‐tyrosine and acetate, with the latter being utilized by *S. cerevisiae* to also form L‐tyrosine. Additionally, the yeast was able to convert this intermediate into the final product by exogenous gene expression, thus achieving a total concentration of 21.16 mg L^–1^ narginine in shake flask at an optimized inoculum ratio of about 70:1 (*S. cerevisiae*: *E. coli*) [95].

Beyond the production of industrially relevant chemicals, microbial consortia could also serve as sources for new drugs. In bacterial‐fungal co‐cultures it has been demonstrated that various secondary metabolites were produced which were not detected in the respective monocultures. These substances mainly seek to subdue the growth of competitors, so they can be considered as potentially useful in pharmaceuticals given their anti‐microbial activities [96, 97].

One very special application of artificial microbial consortia lies in the production of engineered living materials (ELM). One example was reported by Gilbert et al., who designed a co‐culture of *Komagataeibacter rheticus* producing bacterial cellulose and *S. cerevisiae* secreting specific enzymes, which could be incorporated into the cellulose layer within the same process in order to functionalize it. This technology made it possible to develop ELMs able to perceive chemical and optical stimuli and react to them [98].

### 5.2 Artificial photosynthetic co‐cultures

Many microalgae are known to accumulate lipids and are therefore potential candidates for future biofuel production. Co‐culturing these algae with different other organisms can bring advantages as, for example, bacteria supporting lipid formation by provision of nutrients and consumption of oxygen, or filamentous organisms acting as bio‐flocculation agents [99]. Moreover, such co‐cultures might also influence the quality of the biodiesel from the lipids produced as it strongly depends on the percentages of saturated, monounsaturated and polyunsaturated fatty acids [100]. Zhao et al. achieved a significant increase in lipid productivity by co‐cultivating *Chlorella sp*. and *Monoraphidium sp*. as well as a shift in lipid composition towards C18 saturated and monounsaturated fatty acids, which are assumed as advantageous for biodiesel properties [101]. However, many algal co‐cultivation processes are more undefined mixed cultures than defined consortia, as for example described by Boonma et al. [102]. Moreover, challenges like high water and nutrient requirements or low productivities limit the use of microalgal co‐cultures. One possibility for overcoming these challenges may be found in mixed cultures containing microalgae and other microorganisms [103]. Bacteria and algae generally interact in a mutualistic way, with the photosynthetic algae fixating CO_2_ for the supply of carbon source, and the bacteria remineralizing nitrogen, sulfur, or phosphorus, as well as producing co‐factors such as vitamins for algal growth in return [104]. In addition, the other unidentified compounds secreted by bacteria may potentially improve the lipid production of algae [105]. In a study by Xu et al. the lipid accumulation of *Chlamydomonas reinhardtii* was able to be increased 5.9‐fold in a co‐culture with *Azotobacter chroococcum* by using a bacteria‐algae inoculation ratio of 1:40. [106].

Beyond lipids, other products can be produced by photosynthetic bacterial co‐cultures as well. A co‐culture developed by Löwe et al. consisted of two “bio‐modules.” In the first of the latter, the cyanobacterium *Synechococcus elongatus* photosynthetically fixates CO_2_ and converts it into sucrose, which is secreted and serves as carbon source for heterotrophic *P. putida* for intracellular PHA formation. Using a 1.8 L photobioreactor and the delayed inoculation of *P. putida*, a maximum PHA concentration of 156 mg L^–1^ was able to be achieved [107].

Liu et al. established a stable co‐culture of *S. elongatus* with *E. coli* to produce isoprene. Mutualistic interaction (*S. elongatus* photoautotrophically produces oxygen and carbon sources for *E. coli*, which in return delivers CO_2_ and thus lowers oxidative pressure), and a continuous process led to an eightfold increase in isoprene production [108].

In addition to bacteria, other microorganisms such as fungi can also be used within algal co‐cultures. Yang et al. investigated a co‐cultivation approach of *Chlorella sp*. with *Aspergillus sp*. for the improved production of lipids from molasses wastewater. By optimizing the inoculation ratio, a higher lipid content of *Chlorella sp*. and a reduced percentage of polyunsaturated fatty acids leading to potentially better properties as a biodiesel feedstock were achieved, while the fungus improved the substrate supply and decolorization of the molasses wastewater. Additionally, microalgae biomass harvesting was simplified via bioflocculation in the presence of filamentous fungi [109]. The same effect was reported by Wrede et al. in consortia of various microalgae with *Aspergillus fumigatus*, thus offering process advantages [110].

A promising approach for consolidated bioprocesses using microalgae is the combination with oleaginous yeasts. The potential of the latter is based on mutualistic interchanging of carbon and nitrogen sources between the organisms, resulting in enhanced biomass and lipid accumulation. It is mostly the yeast that is responsible for breaking down complex substrates and supplying CO_2_, whereas the photoautotrophic algae fixate CO_2_ and form oxygen and ammonium [103, 111]. One example of these synergistic effects was reported by Yen et al. in a 5 L photobioreactor using the organisms *Rhodotorula glutinis* and *Scenedesmus obliquous*, leading to an increase in lipid content of 60%–70% [112]. Zuccaro et al. also exploited this potential with a co‐culture of the oleaginous yeast *Lipomyces starkeyi* and green microalgae *Chloroidium saccharophilum*. Using a pretreated plant biomass, a lipid content of 0.081 g g^–1^ and an overall productivity of 37.22 mg L^–1^ d^–1^ was achieved in shake flasks [113].

Ling et al. established a co‐culture of *Rhodosporidium toruloides* and *Chlorella pyrenoidosa* in shake flasks by applying domestic and distillery wastewater as carbon and nitrogen sources. The yeast adjusted the pH of the wastewater to a level suitable for growing the microalgae, which were added 40 h after process start. In combination with the removal of inactive biomass after 72 h, a maximum lipid yield of 4.6 g L^–1^ was achieved under nonsterile conditions, as compared to 3.0 g L^–1^ and 3.4 g L^–1^ with algal and yeast monocultures, respectively [114]. Using a consortium of *Rhodotorula glutinis* and *Chlorella vulgaris* in a bubble column photobioreactor, Zhang et al. increased biomass and lipid yields by 17.3% and 70.9%, respectively, in comparison with the monocultures. Synergistic effects were observed regarding the gas balance, substance exchange, dissolved oxygen concentration, and pH level of the co‐culture [115]. Liu et al. established and optimized a co‐culture of *R. glutinis* and *C. pyrenoidosa* regarding the inoculation ratio (1:3) and C/N ratio (64:1), resulting in a lipid yield of 2.48 g L^–1^ and a two‐fold increase in fatty acid productivity compared to the monoculture [116]. Co‐cultures of *S. elongatus* and *R. glutinis* were investigated by Li et al. as an artificial lichen in batch and semicontinuous processes. The biomass and the total lipid yield in the batch co‐culture was 40 ‐ 60% higher than in batch monocultures of the cyanobacterium [117].

Beyond lipids, other, more complex products can also be produced in algae‐yeast consortia by taking advantage of the same principles of gas and substrate exchange. A consortium by Zhang et al. consisting of *R. glutinis* and *C. vulgaris* was used to produce carotenoids from starch wastewater. In an illuminated 5 L bioreactor, the co‐culture produced a maximum total carotenoid concentration of 12.34 mg L^–1^, whereas 8.31 mg L^–1^ were reached in a yeast monoculture cultivation [118].

## 6 CONCLUDING REMARKS

A vast variety of artificial microbial consortia have already been successfully developed within and across taxa. Microbial consortia show huge potential, since beneficial interactions between chosen microbial partners offer the opportunity to use cheap substrates and obtain higher product yields. However, one precondition for engineering a consortium is a knowledge of the interaction mechanisms possible. In future applications, it will be possible to investigate more microorganisms using various screening approaches for generating producing co‐cultures. Through methods like computational modeling and the use of systems biology, it is possible to create co‐cultures in which microorganisms are dependent from each other, benefit from each other, and distribute the metabolic burden of synthetic pathways imposed upon the engineered microorganisms. These methods might also enable the use of a higher number of various microorganisms within one artificial consortium. Therefore, metabolic and enzyme engineering, including methods such as directed evolution, also represent very interesting future directions for the design and improvement of microbial co‐cultures. Nevertheless, many challenges still remain before the successful industrial application of microbial consortia can become widespread across industrial bioprocesses. It should be taken into account that growing a wide variety of microorganisms in a single bioreactor requires both additional effort in expanding the microbial biomass for inoculation at a production scale and more complex control strategies in the production process. The scale‐up of these processes for commercial use also requires integrated technical and economic analyses for evaluating their feasibility. Even though these systems must still be further developed and investigated, co‐cultured bioprocesses represent a promising approach for future applications.

## CONFLICT OF INTEREST

The authors have declared no conflicts of interest.

## AUTHOR CONTRIBUTIONS

Fabian Mittermeier, Prasika Arulrajah, José de Jesús García Lima, Sebastian Hauke, Anna Stock, and Dirk Weuster‐Botz contributed to the conception and design of the review paper; Prasika Arulrajah analyzed the literature with respect to interaction mechanisms; Anna Stock, José de Jesús García Lima, and Sebastian Hauke compiled the literature data about bacterial, fungal and mixed consortia, respectively; Fabian Mittermeier coordinated literature research and evaluation; Prasika Arulrajah, Fabian Mittermeier, and Dirk Weuster‐Botz designed the figures; Fabian Mittermeier, Miriam Bäumler, Prasika Arulrajah, José de Jesús García Lima, Sebastian Hauke, Anna Stock, and Dirk Weuster‐Botz wrote the manuscript. All the authors contributed to the critical revision and final approval of the manuscript.

## Data Availability

Data sharing is not applicable to this article as no new data were created or analyzed in this review article.
